# Beyond Repair Foci: DNA Double-Strand Break Repair in Euchromatic and Heterochromatic Compartments Analyzed by Transmission Electron Microscopy

**DOI:** 10.1371/journal.pone.0038165

**Published:** 2012-05-30

**Authors:** Yvonne Lorat, Stefanie Schanz, Nadine Schuler, Gunther Wennemuth, Christian Rübe, Claudia E. Rübe

**Affiliations:** 1 Department of Radiation Oncology, Saarland University, Homburg/Saar, Germany; 2 Department of Anatomy and Cell Biology, Saarland University, Homburg/Saar, Germany; St. Georges University of London, United Kingdom

## Abstract

**Purpose:**

DNA double-strand breaks (DSBs) generated by ionizing radiation pose a serious threat to the preservation of genetic and epigenetic information. The known importance of local chromatin configuration in DSB repair raises the question of whether breaks in different chromatin environments are recognized and repaired by the same repair machinery and with similar efficiency. An essential step in DSB processing by non-homologous end joining is the high-affinity binding of Ku70-Ku80 and DNA-PKcs to double-stranded DNA ends that holds the ends in physical proximity for subsequent repair.

**Methods and Materials:**

Using transmission electron microscopy to localize gold-labeled pKu70 and pDNA-PKcs within nuclear ultrastructure, we monitored the formation and repair of actual DSBs within euchromatin (electron-lucent) and heterochromatin (electron-dense) in cortical neurons of irradiated mouse brain.

**Results:**

While DNA lesions in euchromatin (characterized by two pKu70-gold beads, reflecting the Ku70-Ku80 heterodimer) are promptly sensed and rejoined, DNA packaging in heterochromatin appears to retard DSB processing, due to the time needed to unravel higher-order chromatin structures. Complex pKu70-clusters formed in heterochromatin (consisting of 4 or ≥6 gold beads) may represent multiple breaks in close proximity caused by ionizing radiation of highly-compacted DNA. All pKu70-clusters disappeared within 72 hours post-irradiation, indicating efficient DSB rejoining. However, persistent 53BP1 clusters in heterochromatin (comprising ≥10 gold beads), occasionally co-localizing with γH2AX, but not pKu70 or pDNA-PKcs, may reflect incomplete or incorrect restoration of chromatin structure rather than persistently unrepaired DNA damage.

**Discussion:**

Higher-order organization of chromatin determines the accessibility of DNA lesions to repair complexes, defining how readily DSBs are detected and processed. DNA lesions in heterochromatin appear to be more complex, with multiple breaks in spatial vicinity inducing severe chromatin disruptions. Imperfect restoration of chromatin configurations may leave DSB-induced epigenetic memory of damage with potentially pathological repercussions.

## Introduction

DNA double-strand breaks (DSBs) generated by ionizing radiation represent an extremely cytolethal form of DNA damage and thus pose a serious threat to the preservation of genetic and epigenetic information. Cells have evolved complex DNA damage response (DDR) mechanisms to ensure genomic integrity that use signaling networks to sense DSBs, arrest the cell cycle, activate DNA repair processes, and, finally, restore the original chromatin structure. Non-homologous end joining (NHEJ) is the predominant DSB repair pathway in higher eukaryotes and operates throughout the cell cycle without the need for template DNA. NHEJ, which essentially mediates direct ligation of broken DNA ends with minimal DNA end processing, is often mutagenic because deletions and insertions can occur at sites of repair [1]. Central to the NHEJ process is the primary recognition of DSBs by the Ku70-Ku80 heterodimer, which creates a preformed ring that sterically encircles free DNA ends without establishing sequence-specific contacts [2]. DNA-bound Ku directs the recruitment of the catalytic subunit of the DNA-dependent protein kinase (DNA-PKcs) via a small helical domain at the C terminus of Ku80, resulting in the assembly of the holoenzyme DNA-PK and activation of its kinase activity. This DNA-PK complex keeps broken DNA ends in close proximity and proper alignment, providing a recruitment platform for subsequent repair factors [1].

Signaling and repair of DNA breaks occur in the context of highly structured chromatin [3]. The fundamental DNA packaging unit of chromatin is the nucleosome, composed of 147 bp of DNA wrapped around a histone octamer. Individual nucleosomes are joined by linker histones such as H1 and further compacted into higher-order chromatin structures by non-histone components, such as heterochromatin protein 1 (HP1). Chromatin compaction acts as a physical barrier to DNA-templated processes such as transcription, and the genome is partitioned into active (euchromatin) and inactive (heterochromatin) domains based on local chromatin fiber density [4].

Emerging evidence suggests that the ability of repair factors to detect DNA lesions and be retained efficiently at breaks is determined by histone modifications around the DSBs and involves chromatin-remodeling events [5]. The most prominent DNA damage induced histone modification is the phosphorylation of the C-terminal tail of H2AX. Phosphorylated H2AX (γH2AX) seems to function as a platform to attract and retain repair proteins, such as MDC1 and 53BP1. The recruitment and accumulation of repair factors at sites of DNA damage results in the formation of radiation-induced foci (RIF). The visualization of RIF by fluorescence microscopy has been used extensively to quantify radiation-induced DSBs and elucidate DNA damage signaling and repair pathways [6], [7], [8], [9], [10], [11], [12]. However, while phosphorylated DNA-PKcs (pDNA-PKcs) forms RIF co-localizing with γH2AX [13], other core members of the NHEJ pathway such as the Ku70-Ku80 heterodimer do not visibly accumulate in RIF because they are only required at low copy number.

Recently, we established a gold-labeling technique for identification and localization of different DNA repair components within the cell nuclei of tissue samples using transmission electron microscopy (TEM) [14]. The high magnification and resolution of TEM permits visualization of the intracellular distribution of repair proteins within subnuclear compartments [15]. Intriguingly, we could show that the assembly of γH2AX, MDC1, and 53BP1 complexes occurs exclusively at heterochromatin-associated DSBs. By labeling phosphorylated Ku70 (pKu70), which binds directly to broken DNA ends in preparation for rejoining, this TEM approach permits reliable detection of DSBs in euchromatic and heterochromatic domains.

The importance of chromatin structure in DDR and DNA repair raises the question of whether breaks in different chromatin environments, specifically euchromatin or heterochromatin, are recognized and repaired with similar efficiency, using the same repair machinery. By labeling core components of NHEJ (pKu70, pDNA-PKcs), we used TEM to monitor the formation and repair of actual DSBs in euchromatic versus heterochromatic regions. Co-labeling pKu70 and 53BP1 with specific euchromatin- and heterochromatin-associated marks in various mouse tissues and human fibroblasts characterized by different patterns of chromatin distribution, we confirm our previous findings that pKu70 allows the detection of euchromatic and heterochromatic DSBs, while 53BP1 marks only the heterochromatic DSBs. Moreover, we extend our previous data by investigating additional very early and late repair-times demonstrating that euchromatic DSBs are verifiable without any delay and rejoined within the first hour after radiation exposure, while heterochromatic DSBs become detectable at later time-points and are repaired with significantly slower repair kinetics, suggesting that densely compacted heterochromatin decelerates DSB processing. Analyzing the molecular composition of persisting DNA lesions, we observed pKu70 clusters of 4 and ≥6 beads in heterochromatin at 48 h and 72 h post-irradiation, likely reflecting multiple DSBs in close proximity. All 4- and ≥6- bead pKu70 clusters co-localize with pDNA-PKcs and disappear within 1 week after irradiation, suggesting that these complex DNA lesions are eventually repaired. Importantly, our data presented here indicate that huge 53BP1 clusters at late repair-times, by contrast, do not co-localize with pKu70 or pDNA-PKcs, suggesting that these persisting 53BP1 agglomerations may not represent actively processed DSBs, but instead mark an incomplete or incorrect restoration of chromatin structure. These results thereby extend our previous understanding of the choreography of the DSB repair process and provide new insights into the dynamic events of the NHEJ pathway progression in the context of the higher-order structure of chromatin.

## Materials and Methods

### Animal Irradiation and Tissue Sampling

Adult C57BL/6 (C57BL/6NCrl) mice (Charles River Laboratories, Sulzfeld, Germany) received whole-body irradiation with 1Gy, 2Gy, 4Gy, 6Gy, 8Gy, or 10Gy (linear accelerator: 6MV-photons; dose rate: 2Gy/min). For dose correlation, C57BL/6 mice were analyzed 5****min and 40****min post-irradiation. For time correlation, different mice were analyzed at 5****min, 20****min, 40****min, as well as 5 h, 24 h, 48 h, and 72 h after irradiation with 6Gy. Three sham-irradiated mice served as controls. After anesthesia, tissues (brain, intestine, skin) were immediately removed and placed in fixative. The experimental protocol was approved by the Medical Sciences Animal Care and Use Committee of the University of Saarland.

### Irradiation of Human Fibroblasts

Dermal fibroblasts were grown in Fibroblast Growth Medium (PromoCell, Heidelberg, Germany) in a humidified 5% CO atmosphere at 37°C. Confluent fibroblasts were irradiated in flasks using an x-ray machine (90 kV, 25 mA, dose-rate: 1.2 Gy/min, 2 mm aluminium filter). After a 40-min repair period at 37°C fibroblasts were trypsinized (0.05% Trypsin/EDTA, Biochrom, Berlin), washed and covered by fixation solution.

### TEM Analysis

Fixed tissues and cells pellets were dehydrated in increasing concentrations of alcohol and infiltrated with LR Gold resin™ (EMS, Hatfield, PA), embedded in fresh resin and stored at –20°C with ultraviolet light illumination. Ultrathin sections were cut on Ultracut UCT Leica™ (Diatome; Biel, Switzerland), picked up with pioloform-coated nickel grids, and processed for immune-labeling. Afterward, sections were incubated with the primary antibody (anti-pKu70 [phosphoSer6], anti-H3K9ac, anti-H3K9me3, Abcam Inc, Cambridge, MA; anti-pDNA-PKcs (phosphoThr2609), Novus Biologicals, Littleton, CO; anti-53BP1, anti-γH2AX [pSer139], Bethyl Laboratories, Montgomery, TX) overnight at 4°C. After rinsing, goat anti-rabbit secondary antibody conjugated with 6-nm or 10-nm gold particles (EMS) was applied to the grids for 1 h. Subsequently, sections were rinsed, fixed with 2% glutaraldehyde, and stained with uranyl-acetate and examined in a Tecnai Biotwin™ transmission electron microscope (FEI Company, Eindhoven, Netherlands). For quantitative analysis, single beads and cluster of beads were counted in cross-sections of 50 different nuclei (≈10 µm in diameter) of cortical neurons, and measurements were extrapolated to the entire round-shaped nucleus.

## Results

**Figure 1:**
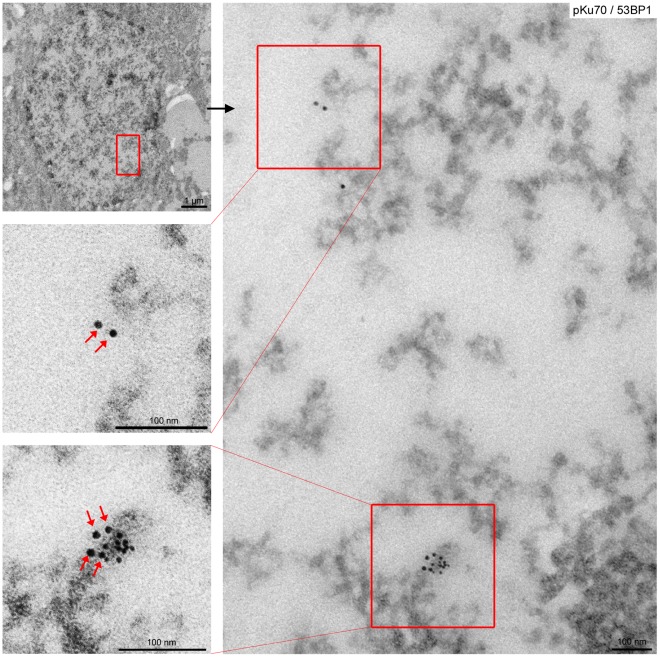
Gold-labeled pKu70 and 53BP1 in cortical neurons of brain analyzed 40 min after irradiation with 6Gy. TEM micrographs of double-labeling of pKu70 (10-nm beads) and p53BP1 (6-nm beads) at different magnifications (boxed regions are shown at higher magnifications in the following images). Complex pKu70 clusters (consisting of 4 gold beads) co-localizing with 53BP1 were observed in heterochromatic regions, but only isolated pKu70 clusters without any p53BP1 binding were observed in euchromatic regions (pKu70 beads are marked by red arrows).

We previously established a gold-labeling technique for electron microscopic identification and localization of different DNA repair factors in cells of irradiated mouse tissue. Here, we monitored the formation and repair of DSBs in the parietal cortex of the mouse brain by quantifying pKu70 clusters in sections of round-shaped cortical neurons. Previous studies have shown that DNA-PKcs phosphorylates serine 6 of Ku70 in the amino- and carboxy-terminal domain close to the DNA-binding canal of the Ku heterodimer, suggesting that DNA-PKcs-mediated phosphorylation of Ku is important for the activation and/or regulation of NHEJ [16], [17]. Using the phosphospecific antibody to serine 6 of Ku70 for immunogold-labeling experiments in specimens analyzed at defined time-points after irradiation with different doses, pKu70 clusters were detected in euchromatic (electron-lucent) and heterochromatic (electron-dense) regions (Fig. 1). Most of the pKu70 clusters in euchromatin consisted of two beads separated by nearly constant distance, presumably reflecting the two pKu70 proteins bound to double-stranded DNA. In heterochromatin, however, pKu70 formed clusters of 2, 4, or ≥6 beads which localized predominantly to the periphery of heterochromatic domains, characterized by more relaxed chromatin structure (light grey regions in TEM) rather than to the tightly packed heterochromatin (dark grey regions).

**Figure 2:**
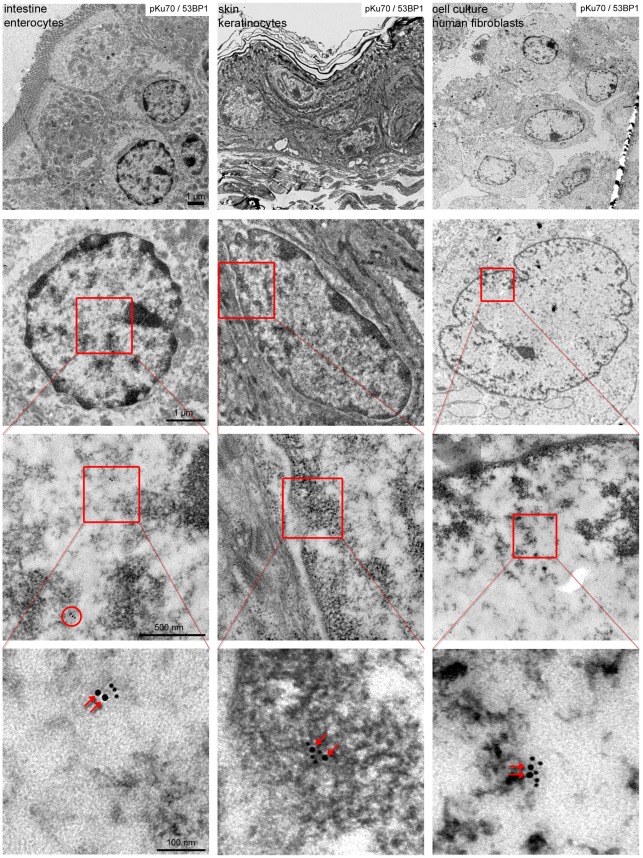
Gold-labeled pKu70 and 53BP1 in mouse tissues and human fibroblasts analyzed 40 min after irradiation with 6Gy. TEM micrographs of double-labeling of pKu70 (10-nm beads) and p53BP1 (6-nm beads) at different magnifications. In enterocytes of small intestine, keratinocytes of skin as well as in human fibroblast, radiation-induced pKu70 clusters (consisting of 2 gold beads, red arrows) co-localize with 53BP1 only in heterochromatic regions.

After labeling pKu70 and 53BP1 with gold beads of different size, we observed a consistent co-localization of pKu70 and 53BP1 in heterochromatic regions, but only pKu70 clusters (without 53BP1 detected) in euchromatic regions (Fig. 1). Similar results were observed after co-labeling of pKu70 and either MDC1 or γH2AX, as shown in the supplemental Fig. S1. To validate our experimental findings in other mouse tissues, we established the co-labeling of pKu70 and 53BP1 in enterocytes of small intestine and epidermal keratinocytes of skin, characterized by more compact heterochromatin, particularly in the periphery of their nuclei. In these tissue samples analyzed 40****min after exposure to 6Gy we observed pKu70 clusters in euchromatic and heterochromatic compartments, but only the pKu70 clusters in heterochromatin co-localized with 53BP1 (Fig. 2).

To further test our hypothesis in human cells, we established the dual labeling of pKu70/53BP1 in human fibroblasts and measured pKu70 clusters before and after *in-vitro* irradiation with different doses. Similar to our data obtained for mouse tissues, we observed very low background levels of clustered pKu70 beads in unirradiated human fibroblasts and a dose-dependent increase of pKu70 clusters in the dose range of 1Gy to 10Gy. Again, our TEM studies revealed that foci-forming factors such as 53BP1 were localized only at heterochromatic but not euchromatic DSBs, while pKu70 labeling of DSBs occurred in both chromatin compartments (Fig. 2), suggesting that pKu70 is also a sensitive and specific marker for unrepaired DSBs in human cells.

**Figure 3:**
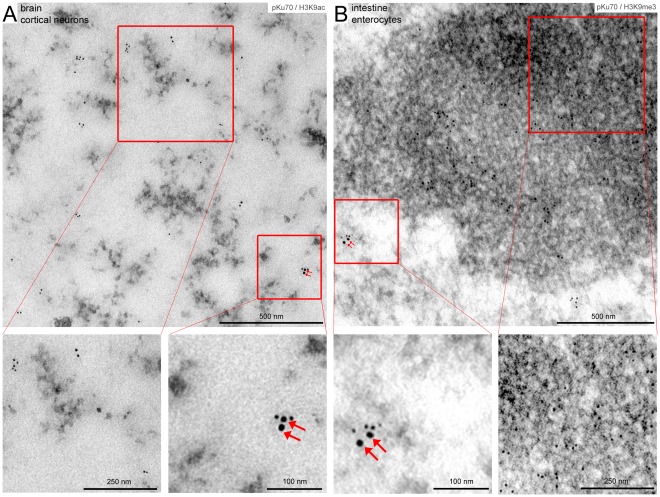
Co-labeling of pKu70 with euchromatic (H3K9ac) and heterochromatic marks (H3K9me3) analyzed 40 min after irradiation with 6Gy. TEM micrographs of double-labeling of pKu70 (10-nm beads) with H3K9ac or H3K9me3, respectively, (6-nm beads) at different magnifications. Immunogold particles directed against H3K9ac are sparsely scattered within electron-lucent euchromatin (**A**), while gold-particles directed against H3K9me3 are highly enriched in electron-dense heterochromatin (**B**). Radiation-induced pKu70 clusters co-localize with H3K9ac (**A**) and H3K9me3 (**B**), suggesting that pKu70 allows the detection of euchromatic and heterochromatic DSBs.

Depending on the level of compaction or state of the chromatin, euchromatic and heterochromatic DNA are characterized by specific patterns of histone modifications, regulating chromatin accessibility and distinct transcriptional functions. Epigenetic hallmarks of silenced or heterochromatic DNA include histone H3 methylation at lysine residue 9 (H3K9me3); open or active euchromatic DNA, by contrast, is associated with acetylated histones such as histone H3 acetylation at lysine residue 9 (H3K9ac). To exclude that in our experimental set-up the preparation of the specimens may deplete loosely bound proteins and/or antibodies from euchromatin, we established the co-labeling of pKu70 with specific euchromatin- and heterochromatin-associated marks. The TEM micrographs in Figure 3 reveal that immunogold particles directed against H3K9ac are sparsely scattered within the electron-lucent euchromatin (Fig. 3A), while gold-particles directed against H3K9me3 are highly enriched in electron-dense heterochromatin (Fig. 3B). This distribution of immunogold complexes directed against euchromatic and heterochromatic histone modifications conform to the higher-order genome organization observed in interphase nuclei, suggesting that the fixation and permeabilization conditions used in this study preserve the chromatin ultrastructure. Using dual gold labeling of pKu70 and H3K9ac or H3K9me3, respectively, after exposure to ionizing irradiation, we observed pKu70 clusters co-localizing with H3K9ac in electron-lucent regions (Fig. 3A), as well as pKu70 clusters co-localizing with H3K9me3 in electron-dense regions (Fig. 3B), confirming that pKu70 allows the detection of euchromatic and heterochromatic DSBs.

Collectively, our results suggest that repair factors such as 53BP1, which are components of RIF as visualized by fluorescence microscopy, appear exclusively in heterochromatic domains, supporting the idea that these factors may promote localized chromatin modifications required for repair in more complex heterochromatin. However, pKu70, an essential alignment factor, must be available at every break and thus appears to be a reliable marker for unrepaired DSBs in euchromatic and heterochromatic domains.

**Figure 4:**
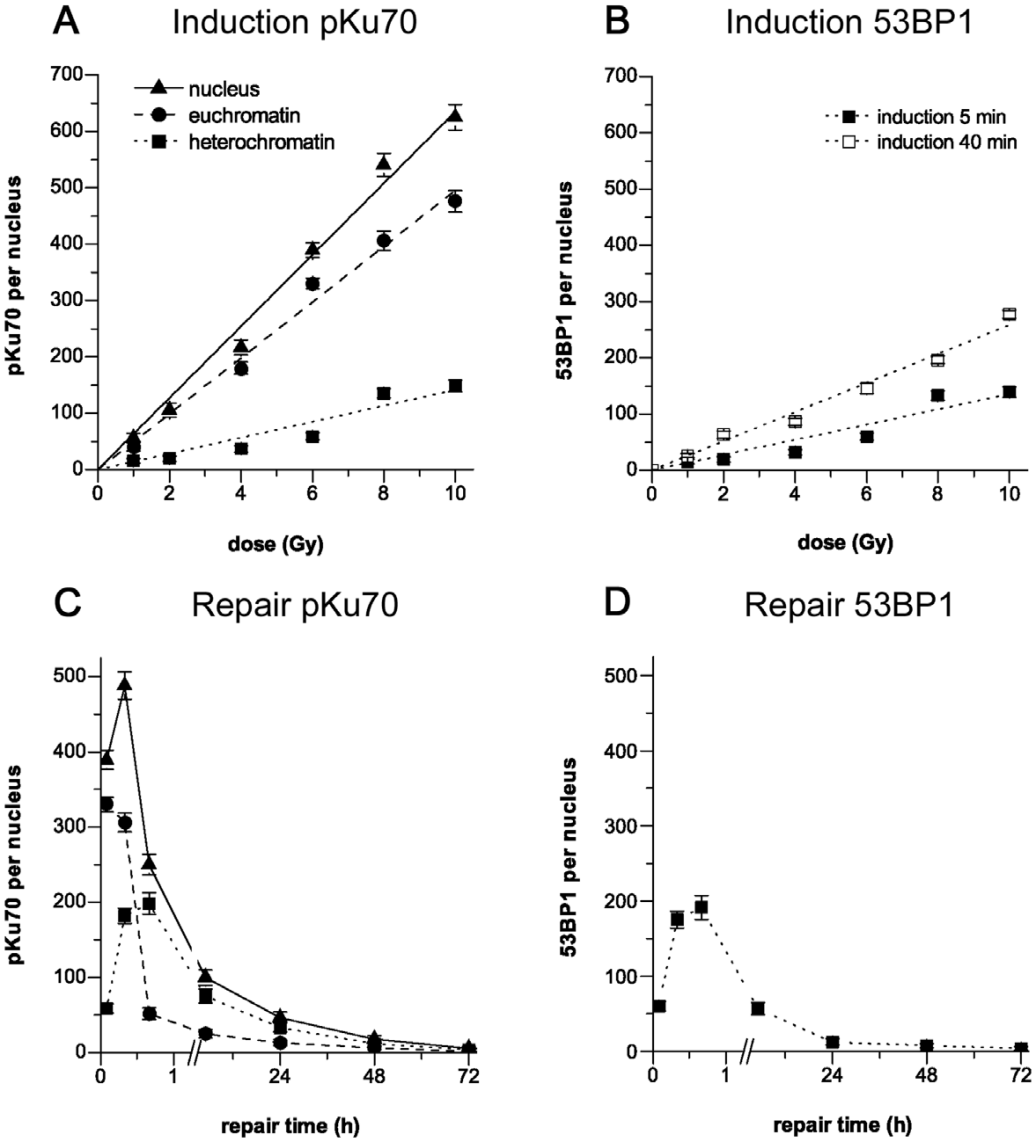
Quantification of gold-labeled pKu70 and 53BP1 in cortical neurons analyzed by TEM. Induction. Quantification of pKu70 and 53BP1 cluster in euchromatic and heterochromatic domains at 5****min and 40****min after irradiation with doses ranging from 1 to 10 Gy. The induction of pKu70 (**A**) and 53BP1 cluster (**B**) was clearly dependent on the radiation dose, with a linear correlation in the dose range of 1 to 10 Gy. **Repair.** Quantification of pKu70 (**C**) and 53BP1 cluster (**D**) per nucleus at 5****min, 20****min, 40****min, 5 h, 24 h, 48 h, and 72 h after irradiation (6Gy). 53BP1 clusters disappeared with kinetics similar to those observed with heterochromatin-associated pKu70 clusters.

In subsequent mouse experiments we analyzed the induction of DSBs in cortical neurons by quantifying pKu70 clusters in euchromatic and heterochromatic domains at 5****min after irradiation with different doses (1–10Gy). Fig. 4A shows that the total number of clustered pKu70 beads is clearly dependent on the radiation dose, with a linear correlation in the dose range of 1 Gy (≈56 clusters/nucleus; extrapolated for the entire nucleus) to 10Gy (≈625 clusters/nucleus) and very low background levels in unirradiated brain tissue (≈4 clusters/nucleus). At 5****min post-irradiation, the number of radiation-induced pKu70 clusters in the heterochromatin was consistently lower (from ≈15 clusters at 1Gy to ≈148 clusters at 10Gy; Fig. 4A) compared to the values obtained for euchromatin (from ≈40 clusters at 1Gy to ≈476 clusters at 10Gy; Fig. 4A) and correlated with the number of 53BP1 clusters indicating exclusively heterochromatic DSBs (≈15 clusters at 1Gy to ≈139 clusters at 10Gy; Fig. 4B). At 40****min post-irradiation, however, we observed higher values for pKu70 and 53BP1 clusters in heterochromatic domains (from ≈26 clusters at 1Gy to ≈278 clusters at 10Gy; Fig. 4B), suggesting that the recognition of DSBs in tightly compacted heterochromatin depends on localized chromatin decondensation, leading to the temporally delayed detection of heterochromatic lesions.

We then analyzed the DSB repair kinetics in euchromatic and heterochromatic domains by quantifying pKu70 and 53BP1 clusters at different time-points after irradiation with 6Gy. In euchromatic subcompartments, the highest value for pKu70 clusters was observed at 5****min (≈330 clusters/nucleus) and subsequently decreased to approximately 306 clusters/nucleus (92%) at 20****min, and approximately 52 clusters/nucleus (15%) at 40****min post-irradiation (Fig. 4C). These data indicate that euchromatin-associated DSBs are promptly sensed and repaired within the first hour after irradiation. In heterochromatic subcompartments, by contrast, the number of pKu70 clusters increased from approximately 59 clusters/nucleus at 5****min to approximately 198 clusters/nucleus at 40****min post-irradiation, and then decreased to approximately 75 clusters/nucleus (38%) at 5 h, approximately 33 clusters/nucleus (17%) at 24h, approximately 12 clusters/nucleus (6%) at 48 h, and approximately 4 clusters/nucleus (2%) at 72 h (Fig. 4C). Similiarly, the number of 53BP1 clusters increased from approximately 60 clusters/nucleus at 5****min to approximately 191 clusters/nucleus at 40****min after irradiation with 6Gy (Fig. 4D), correlating with the time-dependent increase of RIF observed by fluorescence microscopy. Subsequently, 53BP1 clusters disappeared with kinetics similar to the disappearance of the heterochromatin-associated pKu70 clusters, with 57 clusters/nucleus (30%) at 5 h, approximately 13 clusters/nucleus (7%) at 24 h and approximately 4 clusters/nucleus (2%) at 72 h after irradiation (Fig. 4D), suggesting that 53BP1 allows to monitor the repair kinetics of heterochromatic but not euchromatic DSBs. Collectively, these results suggest that DSBs associated with heterochromatin are repaired with slower kinetics compared with DSBs located within euchromatin, presumably because highly compacted chromatin structure poses a physical barrier to the detection of DSBs.

**Figure 5:**
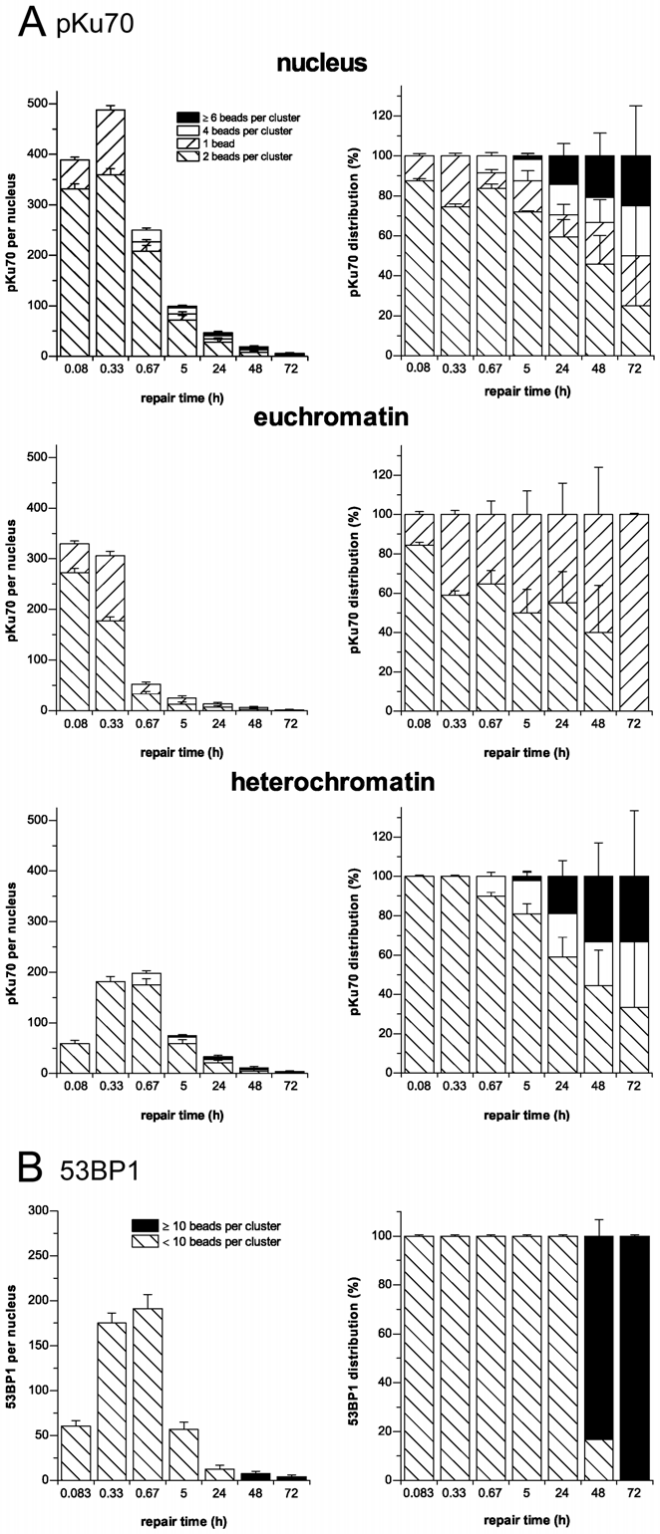
Quantification of the different pKu70 and 53BP1 clusters in euchromatic and heterochromatic compartments of cortical neurons analyzed by TEM. (**A**) Quantification of pKu70 clusters consisting of 1, 2, 4, or ≥6 beads, analyzed separately in euchromatic and heterochromatic compartments at the different time-points after irradiation (6 Gy). We observed solely pKu70 clusters of 1 and 2 beads in euchromatin, but increasingly complex pKu70 clusters with 4 or ≥6 beads in heterochromatin. (**B**) Quantification of 53BP1 clusters <10 and ≥10 beads, analyzed in the heterochromatic compartment at different time-points after irradiation (6Gy). We observed huge 53BP1 clusters at late repair-times.

Although most of the pKu70 clusters consisted of two beads, we also observed some single pKu70 beads in euchromatic domains, particularly at early time-points, and clusters of 4 and ≥6 beads in heterochromatic domains at late repair times. To examine the potential impact of cluster dimensions, we quantified the different clusters of pKu70 (consisting of 1, 2, 4, or ≥6 beads) in euchromatic and heterochromatic regions at different time-points after irradiation (6Gy). In euchromatin compartments we only observed single beads and clusters of 2 beads, with single gold beads perhaps reflecting the high turnover rate of the repair process in euchromatin (Fig. 5A). In heterochromatin, by contrast, we observed a trend toward pKu70 clusters with 4 and ≥6 beads at late repair times (24 h: 40%, 48 h: 55%, and 72 h: 67%), which may reflect multiple breaks in short distance as a result of localized relaxation of formerly highly-condensed heterochromatin (Fig. 5A). For 53BP1, we observed circumscribed clusters of 3–5 gold beads at early time-points, but big clusters of ≥10 gold-beads (up to 30–60 gold beads in huge conglomerations) at late repair-times (48 h and 72 h post-irradiation) (Fig. 5B). While all 53BP1 clusters ≤10 beads (consistently co-localizing with pKu70) disappeared within 48 h post-irradiation, the huge 53BP1 clusters ≥10 beads were detectable even 1 week after radiation exposure (Fig. 5B).

**Figure 6:**
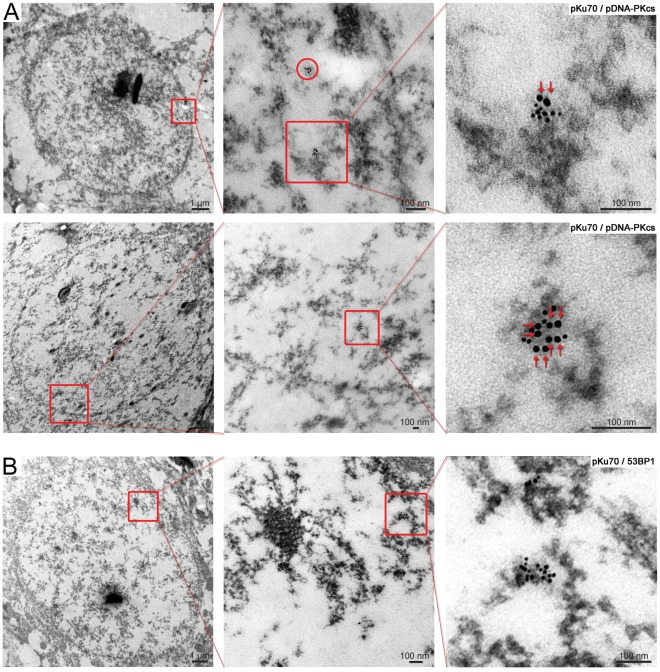
Gold-labeled pKu70 and 53BP1 clusters in cortical neurons analyzed at different time-points after irradiation. TEM micrographs of double-labeling of pKu70 (10-nm beads) and pDNA-PKcs or 53BP1 (6-nm beads) at different magnifications. (**A**) All pKu70 clusters consisting of 2 (upper panel) or more gold beads (lower panel) co-localize with pDNA-PKcs, suggesting that these lesions are actively processed DSBs (analyzed at 20****min and 24 h post-irradiation). (**B**) In contrast, huge 53BP1 clusters in heterochromatin at late repair-times (72 h post-irradiation) do not co-localize with pKu70, suggesting that these lesions do not reflect persistently unrepaired DSBs. (pKu70 beads are marked by red arrows).

To investigate the biological significance of these pKu70 and 53BP1 clusters at late repair-times, we established their co-labeling with phosphorylated DNA-PKcs (pDNA-PKcs). DNA-PKcs is recruited to DSBs by the Ku70-Ku80 heterodimer, with which it forms the holoenzyme DNA-PK that promotes synapsis of the broken DNA ends. For pKu70 we observed a constant co-localization with pDNA-PKcs at the different repair-times, indicating that even the pKu70 clusters of ≥6 beads are actively processed DSBs (Fig. 6A). For the huge 53BP1 clusters at late repair-times, by contrast, we did not observe co-localization with either pKu70 or pDNA-Pkcs (Fig. 6B), suggesting that these lesions may not reflect persistently unrepaired DSBs, but instead mark an incomplete or incorrect restoration of chromatin structure.

Collectively, our results indicate that, while DSBs in euchromatin are immediately sensed and resolved, the detection of DSBs in heterochromatin is protracted due to the time needed to unravel complex chromatin structures at sites of DNA damage. The delayed appearance of complex pKu70 clusters in heterochromatic compartments may reflect multiple breaks in close proximity caused by the direct or indirect effect of ionizing radiation in highly compacted DNA, increasing the chance of disrupting several DNA molecules at once. Moreover, our findings suggest that severe disruptions of chromatin configurations in complex heterochromatin, like those caused by radiation-induced DSBs, may lead to the incomplete or incorrect restoration of the chromatin structure, leaving a DSB-induced epigenetic memory of damage, but not persistently unrepaired DSBs.

## Discussion

The visualization of nuclear foci by fluorescence microscopy has been extremely useful in elucidation of DNA damage signaling and repair pathways. However, recent research providing insights into the chromatin response to DNA damage suggests that most of the molecules of a given factor immobilized near DNA lesions are not directly involved in DNA repair. Chromatin immunoprecipitation experiments revealed that γH2AX is absent from areas directly opposed to DSBs and that the distribution of γH2AX along the chromatin fiber is non-homogenous [18]. Moreover, many proteins that interact with these DNA-damage-modified histones do not directly participate in DNA repair. Instead, spreading of chromatin modifications away from the primary lesions may be an auxiliary mechanism evolved to coordinate repair with transcription and replication (reviewed in [19]). Accordingly, recent discoveries on the mechanisms that govern the formation of DNA repair foci emphasis the importance of not equating such foci with DSBs in all situations [20].

The prerequisite event for all subsequent steps of the NHEJ repair pathway is the initial binding of Ku70/Ku80 heterodimer to DNA ends [21]. Biochemical studies indicate that, once bound to a DSB, Ku recruits the catalytic subunit of the DNA-dependent protein kinase, DNA-PKcs, which stimulates the protein kinase activity of the holoenzyme DNA-PK (reviewed in [22]). The protein kinase activity of DNA-PK is essential for NHEJ and inhibitors of DNA-PK protein kinase activity inhibit NHEJ *in-vitro* and *in-vivo* [23], [24]. Previous studies have shown that DNA-PKcs phosphorylates serine 6 of Ku70, and several amino acids in Ku80, including serines 577, 580 and threonine 715 [16]. Interestingly, these previously identified DNA-PK phosphorylation sites all lie in the unique amino- and carboxy-terminal domains of Ku70/80, that are close to the DNA-binding canal and/or a required for the interaction of Ku with DNA-PKcs [16]. Previous studies therefore suggested, that DNA-PKcs-mediated phosphorylation of Ku could be important for the activation and/or regulation of NHEJ [17]. Using the phosphospecific antibody to serine 6 of Ku70 for immunogold-labeling experiments in specimens analyzed at defined time-points after irradiation with different doses, our TEM studies revealed a linear correlation between pKu70 clusters and irradiation dose. Moreover, these radiation-induced pKu70 clusters disappeared within 72 hours post-irradiation with kinetics previously established for efficient DSB rejoining. Together, these results strongly suggest that pKu70 clusters can be used as a sensitive and highly specific marker for unrepaired DSBs. Using antibodies to non-phosphorylated Ku70, by contrast, we observed huge amounts of single gold-beads scattered in euchromatic and heterochromatic compartments, irrespective of the exposure to ionizing radiation, likely reflecting the non-activated molecules of Ku70 known to be distributed throughout the whole nucleus. However, the precise physiological importance of Ku phosphorylation is not known, and we cannot exclude that there may be breaks that are unlabelled by this phosphospecific antibody.

The interphase nucleus organized into euchromatic and heterochromatic compartments exhibits significant variations in the local DNA concentration [25]. Due to considerable differences in higher-order chromatin structure, it is difficult to determine the relative fractions of the genome packaged in euchromatin and heterochromatin. Moreover, differences in DNA density between euchromatin and heterochromatin likely influence the accessibility of DNA lesions and the speed of their processing. From our data, it is currently not clear whether these pKu70 clusters in euchromatic and heterochromatic domains persist for the same period of time. Extended euchromatin microfibrils offering more accessible DNA surfaces can be probably scanned more efficiently by nuclear factors, therefore favoring fast DNA repair processes. In contrast, highly condensed heterochromatin offering less exposed DNA at its surface, probably requires successive chromatin decondensation to be accessible to the binding of DNA repair factors, leading to the temporally delayed rejoining kinetics. This difference between extended and condensed physical states of the chromatin may explain our observed differences in DSB repair kinetics in euchromatic and heterochromatic compartments.

Interestingly, we observed an increasing amount of 4- and ≥6-bead pKu70 clusters (always divisible by two, reflecting the two pKu70 molecules of the Ku70-Ku80 heterodimer) at later repair times (Fig. 5A). We hypothesize that these complex pKu70 clusters may reflect multiple DSBs in close proximity, caused by the direct or indirect damage of ionizing radiation in highly compacted DNA of heterochromatic regions, and which become detectable after protracted large-scale chromatin relaxation. All 4- and ≥6-bead pKu70 clusters co-localized with pDNA-PKcs (Fig. 6A) and disappeared within 1 week after irradiation, suggesting that these clusters of radiation-induced DSBs were eventually repaired. However, multiple unrepaired DSBs in spatial proximity might be dangerous due to increased likelihood of illegitimate end-joining between broken DNA ends.

For 53BP1, we observed an increase in huge clusters of up to 30–60 gold beads in heterochromatic domains at 48 h and 72 h after irradiation (Fig. 5B), occasionally co-localizing with γH2AX. However, these huge 53BP1 clusters did not co-localize with pKu70 or pDNA-PKcs (Fig. 6B), indicating that these lesions were not actively processed by NHEJ. Moreover, the huge 53BP1 clusters were detectable at nearly constant levels even 1 week after irradiation, suggesting that these lesions may represent permanent chromatin rearrangements due to repair or misrepair of radiation-induced DSBs [19], [26], [27].

TEM detection of labeled pKu70 allowed direct visualization of actual DSBs (not only chromatin-associated epigenetic marks) within the ultrastructure of the nucleus, and revealed different repair kinetics in euchromatic and heterochromatic compartments. Our results provide compelling evidence that chromatin compaction affects the chromatin accessibility at DSBs, suggesting that chromatin complexity rather than DNA damage complexity defines the rate of DSB repair. As soon as heterochromatic DNA lesions are dissected from complex chromatin compaction, they may be processed by NHEJ at the same rate as euchromatic DSBs. Our results are in accordance with the model proposed by P. Jeggo and coworkers that ATM (ataxia telangiectasia mutated) signaling plays a major role in modifying chromatin structure in the vicinity of DSBs and that this ATM function influences the rejoining process of heterochromatic DSBs [28]. Although most DSB repair is ATM independent, approximately 15% of radiation-induced DSBs are repaired with markedly slower kinetics via a process that requires ATM and those mediator proteins, such as 53BP1, that accumulate at RIF. DSBs repaired with slow kinetics predominantly localize to the periphery of heterochromatic domains, suggesting that chromatin complexity and not damage complexity confers slow DSB repair kinetics. ATM’s role in the repair of heterochromatic DSBs involves the direct phosphorylation of KAP-1, a key heterochromatin formation factor [29]. KAP-1 phosphorylation (pKAP-1) arises in both a pan-nuclear and a focal manner after radiation and ATM-dependent pKAP-1 is essential for DSB repair within heterochromatic regions. Mediator proteins such as 53BP1 are expendable for pan-nuclear pKAP-1 whilst being essential for pKAP-1 formation at RIF. These findings suggest that the essential function of 53BP1 is to promote the retention of activated ATM at DSBs, concentrating the phosphorylation of KAP-1 at heterochromatic DSBs, and thereby modulating chromatin structures surrounding the break site. These data conform to our TEM observation that radiation-induced 53BP1 clusters are localized predominantly in the periphery of heterochromatic domains, characterized by more relaxed chromatin structure (light grey region in TEM) rather than to the tightly packed heterochromatin (dark grey regions), suggesting that the local chromatin density may be decreased by 53BP1.

Our findings suggest that after exposure to ionizing radiation nearly all DSBs are efficiently rejoined, sometimes resulting in lasting rearrangements of chromatin, but not leaving persistently unrepaired DSBs. DSBs are an extremely severe form of DNA damage that pose a considerable risk to both genetic and epigenetic integrity. DSB-induced genomic instability and the multistage acquisition of mutations conducive to malignant transformation are frequent hallmarks of cancer. NHEJ, which is commonly employed in response to radiation-induced DNA damage, is known to be mutagenic because ends are processed and joined without a homologous template. Perturbed epigenetic regulation is another essential characteristic of cancer. Severe disruptions of chromatin structure, such as those associated with DSBs, are known to facilitate damage-specific epigenetic responses, potentially resulting in epigenetic regulatory defects with serious implications for gene expression. It is therefore conceivable that persistent, radiation-induced 53BP1 clusters may represent memories of past insults, which could constitute an epimutation transmissible over multiple cell generations [19], [26], [27]. Considering the critical importance of chromatin organization in regulation of gene expression, proper restoration of epigenetic patterns following DSB damage would be crucial to avoid perturbation of transcriptional programs involved in activation or silencing of genes, particularly if these had proto-oncogene or tumor suppressor functions.

Future studies should focus on evaluating the diverse chromatin-remodeling processes involved in DSB repair and whether incomplete or incorrect chromatin remodeling might be associated with DSB-induced epigenetic damage and the perturbation of transcriptional programs.

## Supporting Information

Figure S1 **Gold-labeled pKu70 and γH2AX in cortical neurons of brain analyzed 40 min after irradiation with 6Gy.** TEM micrographs of double-labeling of pKu70 (10-nm beads) and γH2AX (6-nm beads) at different magnifications. pKu70 clusters (consisting of 2 gold beads, marked by red arrows) co-localizing with γH2AX (forming chain-like clusters of gold beads) were observed in heterochromatic regions, but only isolated pKu70 clusters without γH2AX binding were observed in euchromatic regions.
(TIF)
