# Supplementary figures and images for: Beyond Repair Foci: DNA Double-Strand Break Repair in Euchromatic and Heterochromatic Compartments Analyzed by Transmission Electron Microscopy
Source: PLoS One. 2012 May 30;7(5):e38165. doi: 10.1371/journal.pone.0038165 (PMC3364237; doi:10.1371/journal.pone.0038165)

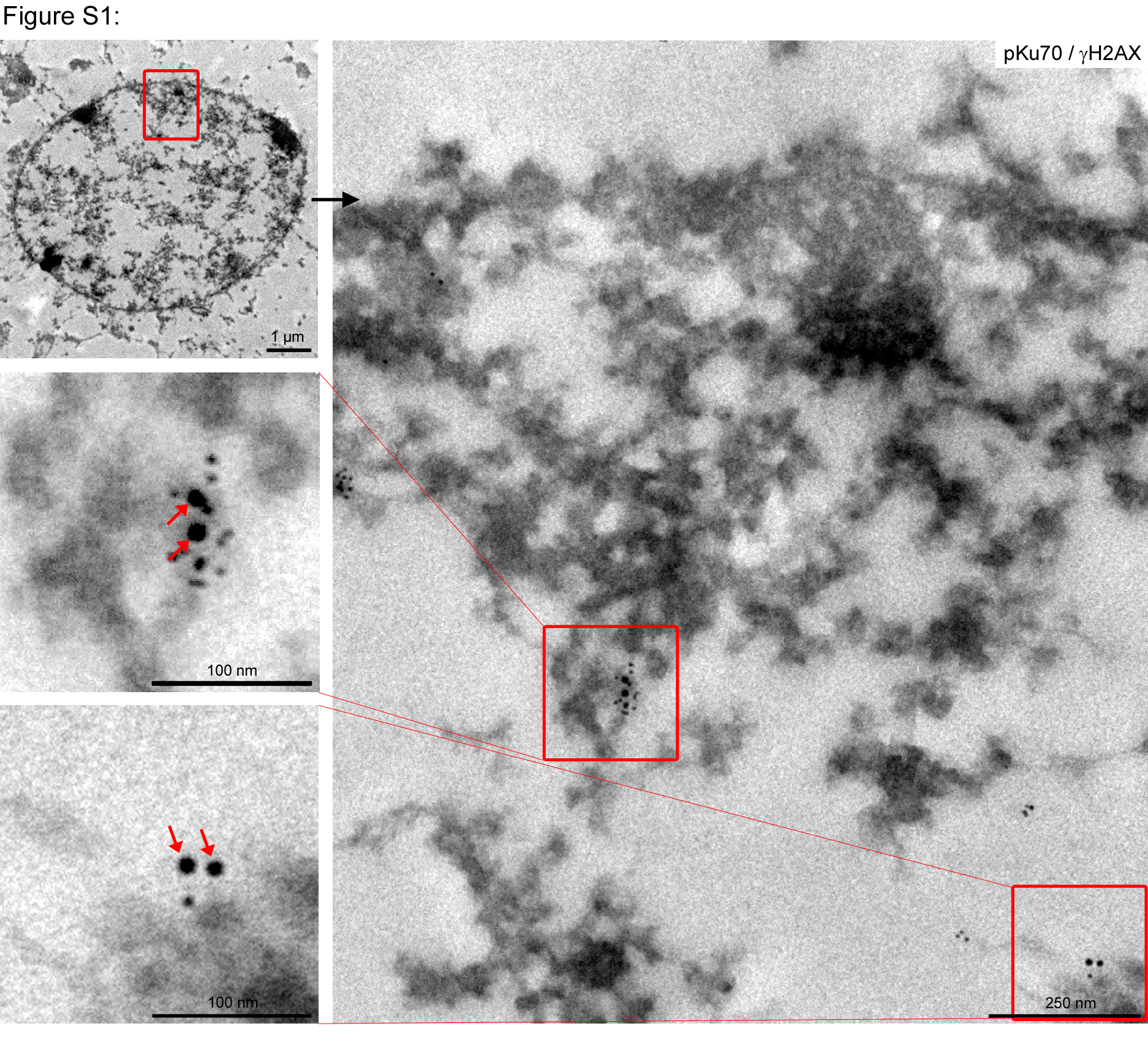

Supplement: Figure S1 — Gold-labeled pKu70 and γH2AX in cortical neurons of brain analyzed 40 min after irradiation with 6Gy. TEM micrographs of double-labeling of pKu70 (10-nm beads) and γH2AX (6-nm beads) at different magnifications. pKu70 clusters (consisting of 2 gold beads, marked by red arrows) co-localizing with γH2AX (forming chain-like clusters of gold beads) were observed in heterochromatic regions, but only isolated pKu70 clusters without γH2AX binding were observed in euchromatic regions. (TIF) [file pone.0038165.s001.tif]
